# Suicidal Ideation Among Children and Young Adults in a 24/7 Messenger-Based Psychological Chat Counseling Service

**DOI:** 10.3389/fpsyt.2022.862298

**Published:** 2022-03-28

**Authors:** Elisabeth Kohls, Lukas Guenthner, Sabrina Baldofski, Melanie Eckert, Zeki Efe, Katharina Kuehne, Shadi Saee, Julia Thomas, Richard Wundrack, Christine Rummel-Kluge

**Affiliations:** ^1^Department of Psychiatry and Psychotherapy, Medical Faculty, Leipzig University, Leipzig, Germany; ^2^Department of Psychiatry and Psychotherapy, University Leipzig Medical Center, Leipzig University, Leipzig, Germany; ^3^krisenchat gGmbH, Berlin, Germany; ^4^Department of Psychology, Chair of Personality Psychology, Humboldt Universität zu Berlin, Berlin, Germany

**Keywords:** suicidal ideation (SI), suicide prevention, chat counseling, adolescent, e-mental health, online intervention, young adults, children

## Abstract

**Background:**

Suicidality in children and young adults is a pervasive problem: approximately 40% of respondents in epidemiological surveys in German schools reported suicidal ideation, while up to 9% reported a suicide attempt in the past. While there is compelling evidence for the effectiveness of telephone-based hotline services, an increasing preference of adolescents for messenger-based counseling services can be observed. Therefore, the present study aims to investigate the utilization behavior and user satisfaction of users contacting a German messenger-based chat counseling service (“*krisenchat*”) regarding suicidal ideation.

**Methods:**

The present cross-sectional study analyzed retrospective anonymous data on sociodemographic variables, utilization behavior, and user satisfaction of *krisenchat* users who used the service between May 2020 and July 2021. Chi-square-tests were used to identify associations of sociodemographic characteristics and utilization behavior with suicidal ideation. Mann-Whitney-*U*-tests were used to compare the user satisfaction and the recommendation-to-others-rate between suicidal and non-suicidal *krisenchat*-users.

**Results:**

In total, chat data of *N* = 11,031 users were collected. Of the *n* = 6,962 users included in the final analysis, *n* = 1,444 (20.7%) contacted *krisenchat* because of suicidal ideation. The average user experiencing suicidal ideation was 17 years old, female and currently not receiving other treatment. Further, suicidal ideation was significantly and positively associated with age and non-suicidal self-injury. Regarding utilization patterns, there were significant positive associations between suicidal ideation and counseling session count, mean amount of messages sent, and mean amount of words used per message by the user. User satisfaction was high, with 64.7% (*n* = 413) of users that answered the feedback survey and experiencing suicidal ideation rating the help they received as at least “good” and a recommendation rate of 89.6% (*n* = 571). Most importantly, no differences were found between users reporting suicidal ideation and those that do not regarding satisfaction and the probability of recommending the service.

**Conclusion:**

Results imply satisfaction with the counseling service among users with suicidal ideation. Nevertheless, there is a need for further research into messenger-based counseling services regarding the prevention of suicidal behavior in children, youths, and young adults. Longitudinal studies are especially needed to assess the effectiveness of messenger-based interventions.

**Study Registration:**

DRKS00026671.

## Introduction

According to the World Health Organization (WHO), suicide is responsible for 1 in 100 deaths worldwide, leading to the deaths of millions of people each year as well as long-lasting effects on the bereaved, such as challenges due to stigmatization and social isolation (1–3). In particular, suicide is the fourth leading cause of death among 15–29 year-olds worldwide and the second or third leading cause of death in adolescents in Europe (4). In Germany, 508 adolescents and young adults (male: 75%, female: 25%) from 10 to 25 years died by suicide in 2020 (5). Consistent with previous studies, girls and women were overrepresented in terms of suicidal ideation or attempts, while deaths by suicide occurred significantly more often in the male population (6–8). Furthermore, previous studies in German students found that up to 40% of students reported suicidal ideation and up to 9% reported suicide attempts (9).

Research on risk factors shows that non-suicidal self-injury (NSSI), psychiatric illness adverse childhood experiences, such as bullying victimization, physical, sexual, and emotional abuse, as well as suicidal bereavement, increase the risk for suicidal ideation or suicide attempts significantly (10–13). Accordingly, suicidality occurs in adolescents with and without underlying psychiatric illness and thus represents a highly relevant health as well as social issue. In line with this argument, the WHO recently published its LIVE LIFE guidance for suicide prevention to curb the number of attempts and deaths by suicide (14). One of the key strategies for suicide prevention outlined in this guide is to foster socio-emotional life skills and mental health literacy in adolescents. This may lead to a reduction in stigma and an increase in mental-health-related knowledge, which may encourage adolescents to seek help. Nevertheless, support and interventions need to be accessible to be able to have an effect on the target groups. The literature indicates that there still is a great need for accessible support and interventions, with 63% of people in Germany with mental health issues aged 18–34 years reporting having never sought help for their issues (15).

A number of barriers hindering the accessibility of mental health-related resources may (in part) explain the low rates of help-seeking found in youths. According to a recent review, such barriers include feared or actual stigmatization, mental health illiteracy, a perceived need for autonomy and self-sufficiency among youths as well as other structural factors such as travel times (16). Additionally, there may be systemic barriers for adolescents with low socioeconomic status, or a diverse background regarding culture or gender, that further limit help-seeking behavior in such populations (17).

As a previous suicide attempt is one of the most important risk factors for suicide in the general population, prevention is of vital importance to mitigate the relative risk of suicide in individuals (18). Unfortunately, research regarding the prevalence of (non-) help-seeking behavior in suicidal or at-risk adolescents and young adults is rather scarce. Nevertheless, the few available studies suggest that help-seeking behavior for suicidality is not common among at-risk adolescents (19, 20). For example, in a sample of 362 European youths, who were considered at-risk for suicidality at a baseline screening and completed a 12-month follow-up self-report, only 45 (12%) sought professional help (20).

Furthermore, a growing number of studies indicate that children, adolescents, and young adults turn to the internet for accessing mental health resources, as it is familiar, easy to access, affords anonymity and may satisfy the perceived need for self-reliance found in some youths and young adults (21–25). Recognizing this trend, a number of crisis hotlines and similar services started incorporating online services, such as chat or e-mail counseling, into their portfolios (26–29). Preliminary evidence suggests the general acceptance and satisfaction of users with those services. While there is not much readily available knowledge regarding the utilization behavior of general users of those services, even less is known regarding subgroups with specific mental health related symptoms like suicidal ideation. Thus, the current investigation focuses on the utilization behavior and user satisfaction of users contacting a German messenger-based chat counseling service (“*krisenchat*”) regarding suicidal ideation.

## Materials and Methods

### Description of *krisenchat*

*krisenchat* (German for “crisis chat”) is a counseling service aimed at children and youths in need of general psychosocial support as well as in acute crisis and can be contacted free of charge, pseudonymously and 24/7 via WhatsApp or SMS. In addition to listening to, calming and comforting users in acute crises, the service engages on cooperative problem solving focusing on promoting users' self-efficacy. If indicated, users are referred to local support services and the health care system. The volunteer counselors have a professional background in health or social work. Counselors are trained to monitor chats for acute suicidality or other acute threats rather than screen for clinical diagnoses. Evidence-based guidelines, including an overview of risk factors and screening questions to address sensitive topics, enable the counselors to assess the current situation and identify individuals at risk. In the case of acute suicidal tendencies, the counselors call an on-call service, staffed by doctors, psychotherapists, psychologists and social workers, which is available 24/7, with whom they discuss further support.

### Participants and Procedure

Anonymized data from all chat users between May 17, 2020 and July 30, 2021 was extracted from the operational database for the purpose of this cross-sectional study. The extracted information included automatically-collected metadata on each chat (e.g., date and time of the first and last contact and total number of messages sent) and information collected and rated by the counselors (e.g., topic of a session). Users were asked to provide their feedback via an automatically generated survey invitation if their chats included at least 30 messages and were not regarded as at-risk of child welfare endangerment by the psychological team. The invitation link to the survey was sent via chat 6 hours after counseling. If users attended more than one session, the survey was only sent to them once after the first chat session. The survey was created using *typeform* in German language. Before participating in the survey, informed consent was retrieved via an opt-in question. Ethical approval was granted by the Ethics Committee of the Medical Faculty, University of Leipzig, on 08-03-2021 (file reference: 372/21-ek).

All in all, data of *N* = 11,031 users were extracted. Out of those, *n* = 7,393 received an invitation to participate in the subsequent survey. Criteria for exclusion were chats marked as “fake chats” by the counselors (*n* = 115, 1.0%; i.e., chats that were started by users without the serious intention of receiving a consultation or without an ongoing crisis), users indicating an age under 6 or over 25 years (*n* = 2,414; 21.9%), no chat topics identified by the counselors (e.g., missing data, or if the addressed concern did not indicate a need for consultation, *n* = 1,539; 14.0%) and missing response by counselors (*n* = 1; 0.0%). In total, the data of *n* = 6,962 users were included in the analysis. The subsequent survey was completed by *n* = 2,762 (39.7%) participants.

### Measures

The current study evaluated utilization behavior and user satisfaction by analyzing automatically collected metadata and data assessed by counselors during or after the sessions. User satisfaction was assessed using information gathered from the feedback survey.

### Suicidal Ideation

According to the ICD-11, suicidal ideation may be defined as any “thoughts, ideas, or ruminations about the possibility of ending one's life, ranging from thinking that one would be better off dead to formulation of elaborate plans” (30). In line with this definition, counselors at *krisenchat* are advised to use the tag *suicidality* during chat counseling to classify suicidal behavior, usually indicated by suicidal ideation or intent, preparatory acts, well-elaborated suicidal plans, suicidal attempts, and other suicide-related behaviors.

### Utilization Behavior

Metadata as well as data on the users collected by counselors during chat sessions were used to evaluate utilization behavior. The metadata included information on the date of first and last contact, the total number of counseling sessions and, finally, the total number of messages and words during the entire consultation period. Data on users' age, gender, and prior or current use of professional help providers were collected and noted by the counselors during the chat, if users disclosed them. The addressed concerns of users were classified into categories by the counselors. Further information, e.g., where the users learned about *krisenchat* (e.g., social media, recommendation) was also asked in the feedback survey.

### User Satisfaction

User satisfaction was assessed as part of the feedback survey using two items. The first item measured user satisfaction on a 5-point Likert scale ranging from 1 = “not at all” to 5 = “very well” by asking the users if the counseling was able to help them with their concerns. The second item asked users how likely they were to recommend the service to others via Net Promoter Scale (NPS; 31), with 0 indicating a 0% probability and 10 indicating a 100% probability of recommending the service to others. As the NPS was developed for marketing purposes, i.e., revenue and company growth, and as there are (to the best of our knowledge) no studies evaluating the psychometric properties of the measure, we elected not to compute the NPS as intended by Reichheld (31) in his original publication. Instead, we elected to interpret the NPS as an indicator of the probability of recommending the service to others on an individual level. Furthermore, according to Reichheld (31), customers or users recommending an organization or service “are also putting their own reputation on the line” (p. 1). As help-seeking for mental health may still be considered a stigmatized issue (16), we elected to use a more liberal cut-off as an indication for recommendation as the original author of the NPS (31). As such, a likelihood of >50% or more was considered as the cut-off to indicate that an individual would be more likely than not to recommend the service to others. Therefore, the likelihood of recommendation was recoded into a binary variable to assess the recommendation rate. Users scoring 6 or higher were assumed to be willing to recommend the service to others.

### Statistical Analysis

Statistical analyses were performed using IBM SPSS Statistics version 27.0. A two-tailed α = 0.05 was applied to statistical testing. Descriptive statistics were performed for sociodemographic variables, utilization behavior, and user satisfaction. Subgroup analyses were conducted using chi-square-tests to identify differences in suicidality regarding different sociodemographic user characteristics. The Standardized Pearson Residuals were used to decompose the effect of significant chi-square-tests (32). To gauge the effect size, the ϕ-coefficent was calculated, while Cramér's V (ϕ_c_) was used when the contingency table was larger than 2x2, with ϕ, ϕ_c_ = 0.10 indicating a small, ϕ, ϕ_c_ = 0.30 an average and ϕ, ϕ_c_ = 0.50 a large effect (33). Because of non-normality of the variables, *Mann-Whitney-U-tests* were used to compare utilization (session count, mean number of messages sent by the user, mean number of words used per message by the user) as well as topic patterns, the user-satisfaction and the recommendation-to-others-rate between *krisenchat*-users experiencing suicidal ideation, and those that did not. Pearson correlations were computed using the z-score of the *Mann-Whitney-U*-test-statistic and interpreted as *r* = 0.10 indicating small, *r* = 0.30 indicating average and *r* = 0.50 indicating large effect sizes (32, 33). Where applicable, Bonferroni Correction was used to account for multiple testing.

## Results

### Sociodemographic Characteristics

A detailed description of sociodemographic characteristics and utilization is displayed in Table 1. Of the *n* = 6,962 users included in the analysis, *n* = 1,444 (20.7%) users contacted *krisenchat* displaying suicidal ideation. Of those who disclosed their gender, 84.4% were identified as female (*n* = 1,065), 13.2% (*n* = 166) as male and 2.5% (*n* = 31) as diverse. The mean user of *krisenchat* who reported suicidal ideation was 17 years old (*M* = 16.50, *SD* = 3.25), female, and currently not receiving treatment (62.4%, *n* = 901). Nearly two thirds (62.0%, *n* = 320) of all users that answered the item asking for professional help-seeking before contacting *krisenchat* and were additionally identified as experiencing suicidal ideation, reported that they had indeed sought professional help prior to contacting *krisenchat*.

**Table 1:** Sociodemographic data and utilization characteristics.

**Variable**		**Suicidal ideation**	**No suicidal ideation**	***X^**2**^***	**ϕ_,_ ϕ_c_**
Gender, *n (%)*				6.41*	0.03
	Female	1,065 (84.4%)	3,923 (71.1%)		
	Male	166 (13.2%)	715 (13.0%)		
	Diverse	31 (2.5%)	78 (1.4%)		
Age groups, *n (%)*				8.80*	0.04
	7–13 yrs.	250 (17.3%)	1,082 (19.6%)		
	14–17 yrs.	745 (51.6%)	2,612 (47.3%)		
	18–25 yrs.	449 (31.1%)	1,824 (33.1%)		
Prior use of professional help services, *n (%)*		320 (62.0%)	877 (15.9%)	23.77***	0.10
Current treatment or intervention, *n (%)*		543 (37.6%)	897 (16.3%)	317.94***	0.21
Time of first contact, *n (%)*				4.85	
	4 a.m.-8 a.m.	56 (3.9%)	225 (4.1%)		
	8 a.m.-12 p.m.	160 (11.1%)	623 (11.3%)		
	12 p.m.-4 p.m.	232 (16.1%)	1,005 (18.2%)		
	4 p.m.-8 p.m.	489 (33.9%)	1,802 (32.7%)		
	8 p.m.-12 a.m.	410 (28.4%)	1,553 (27.8%)		
	12 a.m.-4 a.m.	97 (6.7%)	329 (6.0%)		
				*U*	*r*
Likelihood of recommendation, *M (SD)*		8.55 (2.28)	8.40 (2.45)	662483.50	
	*range*	0–10	0–10		
User satisfaction, *M (SD)*		3.77 (1.07)	3.75 (1.11)	680781.00	
	*range*	1–5	1–5		
Mean session count per user, *M (SD)*		6.03 (9.35)	3.24 (4.97)	2974718.50***	0.18
	*range*	1–141			
Mean number of messages per session, *M (SD)*		28.7 (24.2)	22.68 (17.64)	3256016.50***	0.13
	*range*	3.25–337.00			
Mean word count per message, *M (SD)*	Chat user	15.07 (9.27)	17.46 (11.01)	3432471.00***	−0.10
	*range*	1.59–88.00			

*p < 0.05, ***p < 0.001; X^2^, Chi-Square-Test-Statistic; ϕ, phi-coefficent; ϕ_c_, Cramér's V; U, Mann-Whitney-U-Test-Statstic; r, Pearson correlation coefficient; percentages not adding up to 100% due to missing data.

Subgroup analyses indicated that there were significant associations between age and suicidality, χ^2^_(2)_ = 8.8, *p* = 0.012. An examination of the adjusted Pearson residuals revealed that significantly more individuals in the age group 14–17 were found to be experiencing suicidal ideation than expected, when compared to 7–13 and 18–25-year-olds and correcting for multiple testing. While 18.8% (*n* = 250) of 7–13-year-olds and 19.8% (*n* = 449) of 18–25-year-olds were found to experience suicidal ideation, 22.2% (*n* = 745) of the individuals aged 14–17 were found to experience suicidal ideation.

Furthermore, an investigation of the relative cell frequencies revealed that around 28.4% (*n* = 31) of individuals identifying as diverse, while 21.4% (*n* = 1,065) of users identifying as female and 18.8% (*n* = 166) of users identifying as male reported suicidal ideation. Nevertheless, while gender and suicidality were significantly associated, χ^2^_(2)_ = 6.4, *p* = 0.041, ϕ_c_ = 0.03, an examination of the adjusted Pearson residuals failed to reveal any significant residuals after correcting for multiple testing.

### Utilization Behavior

Descriptive statistics showed that roughly one third of all users experiencing suicidal ideation (*n* = 489, 33.9%) contacted *krisenchat* for the first time between 4:00 and 8:00 pm, while more than a quarter (*n* = 410, 28.4%) sought first contact between 8:00 and 12:00 pm and 10.6% (*n* = 153) contacted *krisenchat* between 12:00 and 8:00 am. This pattern did not differ from users contacting the service with concerns other than suicidal ideation, χ^2^_(5)_ = 4.85, *p* < 0.434.

Results indicate that 44.5% (*n* = 642) of all users experiencing suicidal ideation had more than one topic (*Mdn* = 1) with which they contacted the service of *krisenchat*, while 38.2% (*n* = 2,107) of those not experiencing suicidal ideation shared more than one topic (*Mdn* = 1) during the consultation. When compared, users experiencing suicidal ideation wrote about significantly more topics with counselors, U = 3663908.00, *r* = 0.06, *p* < 0.001; than users who did not suffer from suicidal ideation. Furthermore, significant positive associations were identified for suicidal ideation and NSSI, χ^2^_(2)_ = 407.48, *p* < 0.001, ϕ = 0.24; being in current use of professional help services, χ^2^_(2)_ = 317.94, *p* < 0.001, ϕ = 0.21; being lovesick, χ^2^_(2)_ = 99.70, *p* < 0.001, ϕ = 0.12; and prior use of help services before contacting *krisenchat*, χ^2^_(2)_ = 23.77, *p* < 0.001, ϕ = 0.10.While the topics depression, χ^2^_(2)_ = 36.96, *p* < 0.001, ϕ = 0.07; school, χ^2^_(2)_ = 30.70, *p* < 0.001, ϕ = 0.06; loneliness, χ^2^_(2)_ = 14.64, *p* < 0.001, ϕ = 0.05; pressure because of expectations of others, χ^2^_(2)_ = 18.05, *p* < 0.001, ϕ = 0.05; dealing with mental health issues of others, χ^2^_(2)_ = 17.29, *p* < 0.001, ϕ = 0.05; COVID-19, χ^2^_(2)_ = 15.98, *p* < 0.001, ϕ = 0.05; sexual harassment, χ^2^_(2)_ = 8.25, *p* < 0.01, ϕ = 0.03; anxiety, χ^2^_(2)_ = 7.80 *p* < 0.01, ϕ = 0.03; addiction, χ^2^_(2)_ = 7.73, *p* < 0.01, ϕ = 0.03; and LGBTQIA+, χ^2^_(2)_ = 4.72, *p* < 0.05, ϕ = 0.03; were also identified as being significantly associated with suicidal ideation, the estimated effect sizes were marginal.

Regarding utilization patterns, users affected by suicidal ideation attended on average *M* = 6.0 (*SD* = 9.4) sessions with a mean message count of *M* = 28.7 (*SD* = 24.2) during a single session and a mean word count per message of *M* = 15.1 (*SD* = 9.3). Those not affected by suicidal ideation, on average attended *M* = 3.24 (*SD* = 4.97) sessions, wrote *M* = 22.68 (*SD* = 17.64) messages per session and used *M* = 17.46 (*SD* = 11.00) words per message. In comparison with users not affected by suicidal ideation, those affected by it attended significantly more chat sessions, *U* = 2974718.50, *r* = 0.18, *p* < 0.001; sent on average more messages during a session, *U* = 3256016.50, *r* = 0.13, *p* < 0.001; and had a lower mean word count within a message, *U* = 3432471.00, *r* = −0.10*, p* < 0.001.

### User Satisfaction

In total, *N* = 633 users experiencing suicidal ideation completed the survey on user satisfaction after the counseling. On average, two thirds of all users experiencing suicidal ideation (*n* = 413, 64.7%) were satisfied with the counseling service of *krisenchat*, indicating the service helped them with their concerns “well” or “very well.” Furthermore, the computed recommendation rate was 89.6% (*n* = 571) among those experiencing suicidal ideation. Considering users not experiencing suicidal ideation, 64.6% (*n* = 1,385) reported the service helped them “well” or “very well,” while 87.9% (*n* = 1,878) indicated that they would recommend the service to others. As such, there were no significant differences in satisfaction with the service, *U* = 680781.00, *p* = 0.87, nor regarding the probability of recommending the service to others, *U* = 662483.50, *p* = 0.27, between users experiencing and those that did not experience suicidal ideation.

## Discussion

### Principal Results and Comparison With Prior Work

The present study shows that adolescents *do* contact chat-based counseling services for support in case of suicidal ideation. We found that about one in five users that contacted *krisenchat* was reported to experience suicidal ideation. As such, it can be concluded that suicidality is a very present topic among children and young adults aged between 7 and 25 years, potentially even more in the ongoing pandemic situation. Users experiencing suicidal ideation attended significantly more sessions and wrote more but shorter messages when compared to users not experiencing suicidal ideation. Most importantly, user satisfaction, as well as the likelihood to recommend the service to others, were not significantly different between the two aforementioned groups.

In line with our previous analysis (34), a high satisfaction rate and a high likelihood to recommend the service to others of nearly 90% were found. These results indicate the high acceptability and feasibility of such online services. Further, they suggest the important role such online services may play in early prevention of suicidality (35). As of now, this potential seems to be at least partially untapped, as is indicated by the relative lack of such services in some countries like Germany.

According to previous research, users who seek help online due to suicidal ideation are more likely to be female, tend to use helplines later in the day or at night, and spend more time on the phone (17, 27). The average user affected by suicidal ideation identified in this analysis show consistent characteristics as described in previous literature. Furthermore, we found that roughly 21% of users contacting *krisenchat* reported suicidal ideation. Similarly, *Crisis Text Line*, a U.S.-based counseling service, reports (at the time of writing) that around 20% of users report suicidal thoughts (36). Sindahl et al. (35) report that 7% of users contacting *BørneTelefonen*, a Danish national child helpline, contact the service because of suicidal ideation. While the difference between the *BørneTelefonen* sample and the present sample regarding the proportion of users reporting suicidal ideation might be due to Sindahl et al. (35) analyzing a sample of SMS-based counseling sessions, a definite conclusion cannot be drawn from the available data.

Present results indicate that users experiencing suicidal ideation take part in more chat counseling sessions with significantly more messages with fewer words than non-suicidal users, i.e., they tend to write many short messages during a chat session. A reason for this finding could be that suicidal users of helplines are significantly more likely to reconnect with the helpline than non-suicidal users (37, 38). Another reason for the identified messaging pattern may be motivated by the counselor. Especially if they suspect a user to be at-risk for suicidal behavior, counselors may ask clarifying questions to assess the users' situation, which may prompt short answers such as “yes” or “no.” Previous findings also show that adolescents who are not thriving prefer texting significantly more than their peers, because for some typing is easier than verbally expressing serious concerns (29, 39). Further, helplines should be aware of their function as a potential emotion regulator, which may hinder seeking more effective help, e.g., offline professional help services (35, 37). It also raises the question whether these users demand more of the counselors' capacity because of their high frequent use, as previous studies on telephone helplines have indicated in the past (40, 41).

The identified significant associations between suicidal ideation and NSSI, the affectedness by more than one concern, the current or past use of professional help services were significant but small. Regarding the further literature, suicidality and NSSI are known to be strongly related (42), and in some cases NSSI is a risk behavior for suicidality (10). Other findings also add bullying victimization, loneliness, and problems with parents as important risk factors for suicidal ideation (35). Chat helplines can bridge the gap of perceived lack of parental or social support (39).

However, not all adolescents affected by suicidality are seeking help nor using low-threshold, anonymous helplines. While the data show that every fifth user of *krisenchat* turns to the counseling service because of suicidal ideation, almost 40% never had had contact with the health care system before. Access to primary care for young people, especially for suicide prevention or crisis intervention, remains problematic and needs to be improved. Further, nearly 13% of the users who contacted *krisenchat* because of suicidal ideation were male. This may be explained by the general reluctance of boys or men to seek help (43), which is evident in the underrepresentation of boys and men in suicide or general helplines (44–46). Thus, reaching out to the male population regarding their mental health and supporting them in seeking help remains an open concern. Furthermore, it becomes clear that 2.5% of all *krisenchat* users identify themselves as gender diverse, but suicidality is a concern of nearly 30% within the users of the LGTBQIA+ community. Previous investigations found that persons from the LGTBQIA+ community seek out specialized LGTBQIA+ helplines and are less likely to utilize general helplines. This indicates that in addition to general help-seeking barriers, there may be systematic barriers in engaging youth from culturally or gender diverse backgrounds, e.g., because of the fear of shame and stigma (47).

On the part of the providers, there are several variables that may also influence the counseling process. For example, reduced stigma toward depression and literacy about suicidality are associated with confidence in exploring suicidality or risk-factors associated with suicidality (48). However, chat-based counseling, in addition to the numerous advantages, also brings with it some barriers. For example, risk assessment standards are needed for chat communication due to missing informative verbal and mimic cues (29, 49). Additionally, the lack of non-verbal cues aggravates for counselors to establish and maintain a therapeutic relationship, which is known as one of the key factors and of great influence in face-to-face mental health services and its outcomes (50, 51). Also, in some studies, counselors reported higher difficulties and a decreased ability to establish a therapeutic relationship in the digital environment (51, 52). As may be expected for a relatively new counseling setting, research exploring potential barriers and drawbacks of chat counseling is still lacking.

### Strengths and Limitations

The present study is the first study to examine the utilization behavior and satisfaction with a chat-based crisis counseling service in German speaking children, teenagers, and young adults reporting suicidality using real world data. However, several limitations need to be taken into account. As we employed a retrospective study design, the present study relied on convenience sampling. As such, the resulting sample is not representative of the general population. Furthermore, no standardized measurement instruments were employed and the present study did not supply follow-up data. Such longitudinal data would be of great use to gauge the success and effectiveness of *krisenchat* regarding, e.g., successful referrals to professional mental health services or the alleviation of suicidal ideation.

## Conclusion

As the results of the present study show, the high level of satisfaction with the low-threshold messenger-based chat counseling service that was previously found for a general sample of users (34) also extends to users experiencing suicidal ideation. Therefore, it may be concluded that young people use the internet to access anonymous chatting, help-seeking, and crisis intervention services for serious concerns like suicidality. Expanding on this, our results imply that users use the services of *krisenchat* during all hours of the day (and night). As such, existing telephone-based counseling services may want to consider expanding their services to messenger and internet-based modalities and to all hours of the day to reduce barriers and facilitate access to their services, while political stakeholders should ensure that resources are provided for the creation of new and the expansion of already existing services. While the present and previous studies show that users of messenger-based crisis intervention services are highly satisfied with the services, further research (in particular longitudinal studies) is needed to explore the effectiveness of such services regarding the prevention of suicides and other outcomes of interest. Moreover, longitudinal studies may help to investigate the underlying processes of successful suicide prevention interventions using messenger and chat services, as well as to identify modifiable protective factors.

## Data Availability Statement

The raw data supporting the conclusions of this article will be made available by the authors, without undue reservation.

## Ethics Statement

The studies involving human participants were reviewed and approved by University Leipzig, Medical Faculty. Written informed consent from the participants' legal guardian/next of kin was not required to participate in this study in accordance with the national legislation and the institutional requirements.

## Author Contributions

EK, LG, and CR-K designed the study. ZE, LG, and SB performed the statistical analysis. EK and LG drafted the article. SS, JT, and RW prepared the data set and edited previous versions of the manuscript. ME, ZE, SB, EK, KK, and CR-K discussed the results and contributed to the final manuscript. All authors have approved the final manuscript.

## Funding

This work was supported by The Federal Ministry of Health (ZMI1-2521FEP001).

## Conflict of Interest

CR-K received lecture honoraria from Recordati and Servier outside and independent of the submitted work. ME, SS, JT, and RW were employed by krisenchat gGmbH. The remaining authors declare that the research was conducted in the absence of any commercial or financial relationships that could be construed as a potential conflict of interest.

## Publisher's Note

All claims expressed in this article are solely those of the authors and do not necessarily represent those of their affiliated organizations, or those of the publisher, the editors and the reviewers. Any product that may be evaluated in this article, or claim that may be made by its manufacturer, is not guaranteed or endorsed by the publisher.
